# Association of continuous renal replacement therapy downtime with fluid balance gap and clinical outcomes: a retrospective cohort analysis utilizing EHR and machine data

**DOI:** 10.1186/s40560-024-00772-w

**Published:** 2024-12-31

**Authors:** Chloe Braun, Tomonori Takeuchi, Josh Lambert, Lucas Liu, Sarah Roberts, Stuart Carter, William Beaubien-Souligny, Ashita Tolwani, Javier A. Neyra

**Affiliations:** 1 The University of Alabama at Birmingham, Birmingham, AL USA; 2 Tokyo Medical and Dental University, Tokyo, Japan; 3 University of Cincinnati, Cincinnati, OH USA; 4 Public Health Sciences Division, Fred Hutchinson Cancer Center, Seattle, WA USA; 5 Centre Hospitalier de L’Universite de Montreal, Montreal, QC Canada

**Keywords:** Fluid management, CRRT, Mortality, AKI, Downtime

## Abstract

**Background:**

Fluid balance gap (FBgap—prescribed vs. achieved) is associated with hospital mortality. Downtime is an important quality indicator for the delivery of continuous renal replacement therapy (CRRT). We examined the association of CRRT downtime with FBgap and clinical outcomes including mortality.

**Methods:**

This is a retrospective cohort study of critically ill adults receiving CRRT utilizing both electronic health records (EHR) and CRRT machine data. FBgap was calculated as achieved minus prescribed fluid balance. Downtime, or percent treatment time loss (%TTL), was defined as CRRT downtime in relation to the total CRRT time. Data collection stopped upon transition to intermittent hemodialysis when applicable. Linear and logistic regression models were used to analyze the association of %TTL with FBgap and hospital mortality, respectively. Covariates included demographics, Sequential Organ Failure Assessment (SOFA) score at CRRT initiation, use of organ support devices, and the interaction between %TTL and machine alarms.

**Results:**

We included 3630 CRRT patient-days from 500 patients with a median age of 59.5 years (IQR 50–67). Patients had a median SOFA score at CRRT initiation of 13 (IQR 10–16). Median %TTL was 8.1% (IQR 4.3–12.5) and median FBgap was 17.4 mL/kg/day (IQR 8.2–30.4). In adjusted models, there was a significant positive relationship between FBgap and %TTL only in the subgroup with higher alarm frequency (6 + alarms per CRRT-day) (β = 0.87 per 1% increase, 95%CI 0.48–1.26). No association was found in the subgroups with lower alarm frequency (0–2 and 3–5 alarms). There was no statistical evidence for an association between %TTL and hospital mortality in the adjusted model with the interaction term of alarm frequency.

**Conclusions:**

In critically ill adult patients undergoing CRRT, %TTL was associated with FBgap only in the subgroup with higher alarm frequency, but not in the other subgroups with lower alarms. No association between %TTL and mortality was observed. More frequent alarms, possibly indicating unexpected downtime, may suggest compromised CRRT delivery and could negatively impact FBgap.

**Supplementary Information:**

The online version contains supplementary material available at 10.1186/s40560-024-00772-w.

## Background

Acute kidney injury (AKI) is common in critically ill adults admitted to the intensive care unit (ICU) [1]. Critically ill patients with severe AKI and positive fluid balance or fluid accumulation that do not respond to diuretics are often treated with continuous renal replacement therapy (CRRT) with the goal of mitigating poor outcomes that are associated with fluid accumulation [2–7]. Managing positive fluid balance with CRRT requires prescription of a fluid removal rate according to fluid balance goals and adjusting these goals based on achieved fluid balance and the patient’s clinical course [8]. It has recently been shown that despite provider best efforts, there is frequently a gap between the prescribed fluid balance and what is effectively achieved. Moreover, this fluid balance gap (FBgap) has been shown to be associated with hospital mortality [9]. There is paucity of data about what contributes to the FBgap. The Acute Disease Quality Initiative (ADQI) 2019 consensus conference identified (among other factors) downtime as a key quality indicator in CRRT treatment [10]. However, the impact downtime has on fluid management and clinical outcomes has not been thoroughly investigated [11–14].

We hypothesized that percent treatment time loss (%TTL), in other words CRRT machine downtime during a patient’s CRRT treatment course, would be positively associated with FBgap—higher downtime, higher FBgap. We aimed to describe how %TTL relates to FBgap and relevant clinical outcomes after adjusting for patient-related factors. Finally, we set out to describe patient and machine factors that may be implicated in %TTL.

## Methods

### Study design and participants

We conducted a single-center, retrospective cohort study of adult patients (≥ 18 years) admitted to all ICUs with AKI who received CRRT at the University of Kentucky Albert B. Chandler Hospital between August 2017 and April 2021. We excluded patients for whom ICU admission and discharge records were unavailable, those missing data (body weight at admission, Sequential Organ Failure Assessment (SOFA) score, fluid balance, and machine parameters), and those with end stage kidney disease or kidney transplant. Body weight values below the 0.5th percentile or above the 99.5th percentile were considered outliers and treated as missing. To address outliers in FBgap, we used Cook’s distance based on a linear regression model that included both FBgap and %TTL as the dependent and independent variable, respectively [15]. See additional materials regarding distribution of FBgap (Additional Fig. 1). Patients with Cook's distance values exceeding four times the mean Cook's distance of the cohort were excluded from the analysis. CRRT was performed with continuous venovenous hemodiafiltration according to institution standards with regional citrate most commonly used for anticoagulation. This study was approved by the Institutional Review Board (IRB) from the University of Kentucky (17–0444-P1G). Given the observational nature of this investigation, informed consent was waived by the Institutional Review Board.

**Fig. 1:**
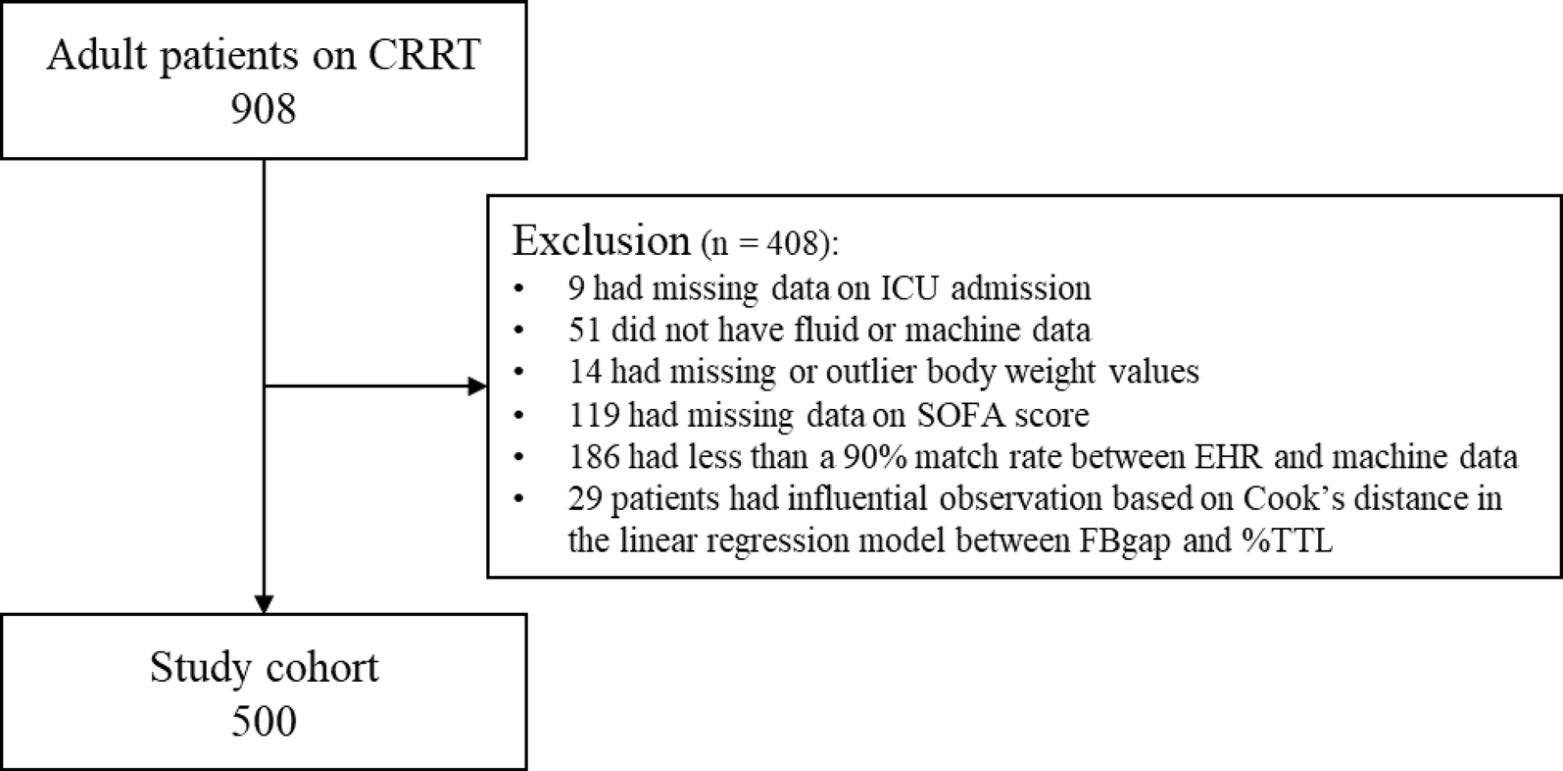
Consort diagram. RRT—continuous renal replacement therapy, ICU—intensive care unit, SOFA—sequential organ failure assessment, EHR—electronic health record, FBgap—fluid balance gap, %TTL—treatment time lost

### Data sources

We utilized two data sources: electronic health record (EHR) and CRRT machine data. We obtained data cards containing machine parameters from the CRRT machines through manual extraction. We matched EHR data with CRRT machine data in a phased approach, utilizing variables in descending order of priority, such as the medical record number, patient name, encounter identification number, CRRT start and stop dates/times, death date/time (if applicable), body weight, and total machine fluid removal per day. For patients who could not be matched through these criteria, we employed a further matching process using the remaining available variables from the CRRT data and dates with information available in the patient record. These variables were cross-referenced against known values in the clinical record. To avoid the use of inaccurate information, only patients whose machine data and EHR matched with a timestamp concordance rate of 90% or higher were included. For a more detailed description of the matching process, refer to the additional material (Additional Table 2).

### Primary independent and dependent variables

The primary independent variable, %TTL, was calculated based on data from CRRT machines. %TTL represents the ratio of CRRT downtime (therapy is paused) to the total CRRT time when the machine is on (except when priming). As %TTL is a ratio, it is adjusted for total time on CRRT. The analysis would have excluded any intentional CRRT stops. Similarly, once CRRT was permanently stopped, such as in patients with kidney function recovery or planned transition to intermittent hemodialysis data collection stopped. Data regarding specific causes of downtime were unavailable with the exception of frequency of CRRT alarms. Alarms from the CRRT machine, a component of downtime calculation, were categorized as catheter alarms (access and return alarms) and filter alarms (transmembrane pressure [TMP], filter pressure [FP], and clotting alarms). The frequency of these alarms, along with the number of filter changes and the effluent flow rate (CRRT dose), were extracted from machine data. The daily average alarms and number of each type of alarm were calculated. Total alarms per day were categorized into three groups: 0–2, 3–5, and 6 + , to ensure a close to equal distribution of patients within each group, guided by prior data distribution knowledge at the study site. Details on alarm processing is available in the additional materials (Additional Table 2).

The primary outcome of interest, FBgap, represents the deviation of the actual net fluid balance from the pre-established fluid balance goal, adjusted by the number of CRRT days and the patient's admission body weight. FBgap was calculated using the formula: (actual patient’s net fluid balance – patient’s fluid balance goal prescribed during CRRT)/total CRRT days/body weight, expressed in mL/kg/day. Additional Fig. 2 illustrates the distribution of patient’s FB prescribed and FB achieved. FB goal was determined by clinicians as "no fluid removal in 24 h", "net negative X liters in 24 h", or "net even matching intake and output in 24 h” and was aggregated during the total period of CRRT for analysis. Notably, if fluid removal was not clinically indicated a FB goal was still documented. Similarly, if a patient had increased native urine output, this would be included in the net FB calculation and thus accounted for in their FBgap. These goals were set according to clinicians' goals of fluid management and adjusted on average once or twice a day. Nevertheless, the FB goal could be adjusted more frequently if clinically necessary as the bedside nurse has a CRRT dedicated flowsheet that compiled all fluid management parameters every hour. This flowsheet accounted for the FB goal per hour (ex. FB goal net negative 50 ml/h for a FB goal net negative of 1.2 L in 24 h), the deficit from the prior hour, and the suggested target to set the machine for the next hour. These data were documented in the electronic CRRT flowsheet and then extracted from the Electronic Data Warehouse for validation and analysis. The FB goals were determined by consultant nephrologists in collaboration with ICU physicians, based on the institutional standard of care.

**Fig. 2:**
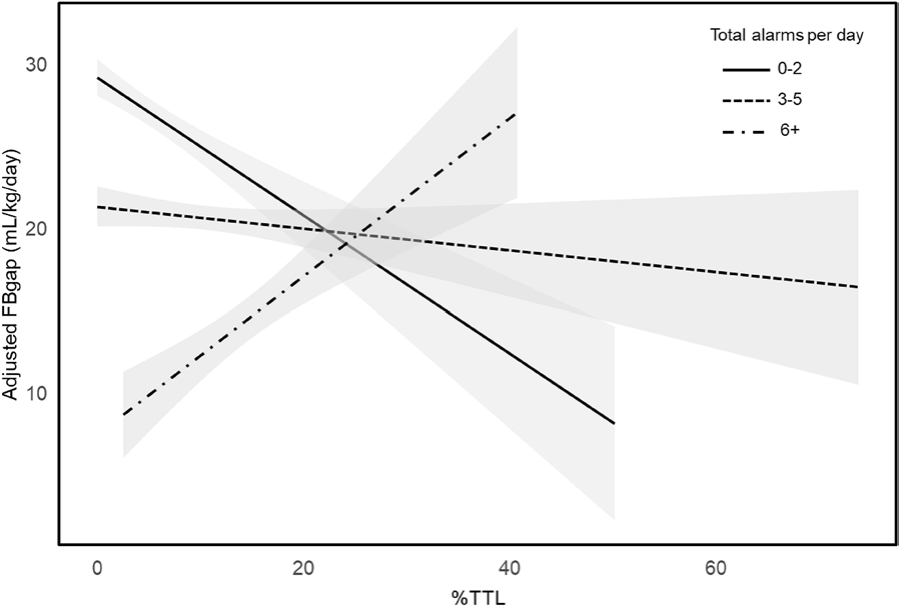
Relationship between FBgap and %TTL stratified by total alarms. FBgap, fluid balance gap; %TTL, percent treatment time loss; CCI, Charlson comorbidity index; SOFA, sequential organ failure assessment; CRRT, continuous renal replacement therapy; MV, mechanical ventilation; IABP, intra-aortic balloon pump; VAD, ventricular assist device; ECMO, extracorporeal membrane oxygenation. Adjusted FBgap was calculated based on the multivariate linear regression model with FBgap as the dependent variable and %TTL, along with age, sex, race, CCI, SOFA score at CRRT initiation, use of MV at CRRT initiation, use of circulatory support devices (any of IABP, VAD, ECMO) at CRRT initiation, and %fluid overload as independent variables

### Other variables: patient characteristics and process variables

Patient demographics (age, sex, race, body weight, survival status, baseline estimated Glomerular Filtration Rate (eGFR), SOFA score [16], and International Classification of Diseases-code-based comorbidities) were extracted from the EHR. The Charlson Comorbidity Index (CCI) was calculated based on recorded comorbidities [17]. The use of CRRT, mechanical ventilation (MV), intra-aortic balloon pump (IABP), ventricular assist devices (VAD), and extracorporeal membrane oxygenation (ECMO) was also extracted from EHR. ICU and device free days were computed as the total number of days without each respective intervention within a 28-day period starting from the initiation of CRRT. If a patient died within 28 days, the free days were counted as zero. Fluid overload (%FO) was defined at CRRT initiation by the following formula: (Total Fluid Input in L – Total Fluid Output in L) *100/Weight (Admission) in Kg.

### Statistical analysis

The analyses in this study were divided into two major parts, and all statistical analyses and figures were generated using R Studio version 4.2.2.

#### Primary analysis

To elucidate the relationship between %TTL and FBgap, a comprehensive linear regression analysis was conducted with multiple model specifications. Specifically, models were fitted both with and without adjustment for patient-specific factors, and with and without identified interactions. To identify these interactions, we utilized the Feasible Solution Algorithm (FSA) [18], which identified potentially novel two-way interactions between %TTL and patient/machine factors that contributed to variations in FBgap. This interaction identification analysis aimed to investigate how the main independent variable (%TTL) differentially affected FBgap across various subgroups, thereby elucidating which group(s) are most susceptible to adverse effects associated with %TTL. In essence, this analysis provides a nuanced understanding of how %TTL influences FBgap in distinct patient and machine contexts, ultimately informing strategies for mitigating potential negative outcomes.

#### Secondary analysis

We reported the characteristics of the cohort by %TTL tertiles, as well as by survival status at discharge. To explore the factors associated with %TTL, we used a linear regression model to examine patient factors, such as age, sex, race, CCI, SOFA score at CRRT initiation, use of MV at CRRT initiation, use of circulatory support devices (including IABP, VAD, ECMO) at CRRT initiation, and %FO at CRRT initiation. We also investigated the association between machine alarms, including access pressure, return pressure, transmembrane pressure, filter pressure, and clotting alarms, with %TTL.

#### Multiplicity

The alpha (α = 0.05) spending in this study relates to the models outlined in the primary analysis section above. In total, the four planned models contained the main independent variable (%TTL) either with or without adjustment, and with or without an interaction term. To address the issue of multiplicity, we used the Bonferroni correction to adjust the significance level for these four models. Within these models, we identified all terms involving %TTL (main or interaction terms). This number of terms, T, was used to adjust the cutoff for statistical significance by dividing 0.05 (α) by the number of terms. We also report the results of exploratory analyses, including logistic regression models for hospital mortality and linear regression models for ICU-, CRRT-, and MV-free days, without adjustments for multiple testing, as they are not part of our alpha spending aims in this study.

## Results

### Cohort characteristics and clinical outcomes

We identified 908 patients admitted to the ICU who received CRRT during the study period. A total of 500 patients accounting for 3,630 patient–CRRT days were included in the final analysis (Fig. 1). The cohort had a median age of 59.5 years (Interquartile range [IQR]: 50.0, 67.3) and consisted of 305 males (61.0%), mostly white (89.8%). The median age differed based on %TTL tertile. The median baseline eGFR was 41.6 mL/min/1.73m^2^ (IQR: 18.5, 74.8), the median CCI was 5 (IQR: 3, 7), and the median SOFA score at CRRT initiation was 13 (IQR: 11, 16) (Table 1). Cohort characteristics by mortality can be found in additional materials (Additional Table 3). The median total alarms per day was 2.67 (IQR: 1.50, 4.72), with catheter alarms being more frequent than filter alarms. The median FBgap was 17.37 mL/kg/day (IQR: 8.22, 30.39). The association between patient and machine factors with FBgap can be found in additional materials (Additional Table 4). The median %TTL was 8.14% (IQR: 4.34, 12.45). The overall mortality was 54.2%. Median ICU length of stay was 10 days (IQR: 5, 19) and the median CRRT duration was 5 days (IQR: 3, 9) (Table 2).

**Table 1:** Cohort characteristics stratified by %TTL tertiles

Patient factors		Overall (*n* = 500)	%TTL lowest tertile* (*n* = 167)	%TTL middle tertile* (*n* = 166)	%TTL highest tertile* (*n* = 167)
Age (years)	Median [IQR]	59.5 [50.0, 67.3]	62.0 [51.5, 71.0]	59.0 [51.3, 66.0]	57.0 [44.0, 65.0]
Sex, male	*n* (%)	305 (61.0)	100 (59.9)	106 (63.9)	99 (59.3)
Race	*n* (%)				
Black		39 (7.8)	11 (6.6)	10 (6.0)	18 (10.8)
White		449 (89.8)	152 (91.0)	151 (91.0)	146 (87.4)
Other or unknown		12 (2.4)	4 (2.4)	5 (3.0)	3 (1.8)
Body weight at ICU admission (kg)	Median [IQR]	93.7 [78.4, 113.0]	97.5 [83.6, 113.6]	92.7 [77.2, 114.5]	91.0 [76.0, 110.5]
Charlson comorbidity index	Median [iqr]	5 [3, 7]	5 [3, 7]	4 [2, 6]	5 [3, 7]
SOFA score at CRRT initiation	Median [iqr]	13 [11, 16]	14 [11, 16]	13 [11, 16]	13 [11, 15]
SOFA score at ICU admission	Median [iqr]	12 [9, 14]	12 [9, 14.50]	11 [9, 14]	12 [8.50, 14]
Baseline eGFR (mL/min/1.73m2)	Median [iqr]	41.6 [18.5, 74.8]	35.0 [18.2, 63.5]	48.4 [19.8, 80.5]	42.3 [17.7, 74.4]
Mechanical ventilation at CRRT initiation	N (%)	404 (80.8)	131 (78.4)	142 (85.5)	131 (78.4)
Cardiac support device at CRRT initiation	N (%)	52 (10.4)	18 (10.8)	21 (12.7)	13 (7.8)
Time from ICU admission to CRRT initiation (days)	Median [iqr]	1 [1, 4]	1 [1, 3]	1 [1, 4]	2 [0, 5]
%Fluid overload from ICU admission to CRRT initiation	Median [iqr]	0.92 [0, 4.67]	0.66 [0.00, 3.32]	0.92 [0.00, 5.18]	0.95 [0.00, 7.23]
Machine factors†					
Total alarms	Median [IQR]	2.67 [1.50, 4.72]	1.40 [0.60, 2.28]	2.81 [1.76, 4.03]	4.69 [2.93, 8.00]
Catheter alarms	Median [IQR]	2.43 [1.29, 4.12]	1.25 [0.50, 2.00]	2.61 [1.67, 3.62]	4.29 [2.38, 6.73]
Access alarms	Median [IQR]	1.25 [0.50, 2.28]	0.50 [0.00, 1.14]	1.41 [0.76, 2.23]	2.08 [1.24, 3.50]
Return alarms	Median [IQR]	1.00 [0.43, 1.80]	0.50 [0.00, 1.00]	1.00 [0.50, 1.65]	1.55 [0.92, 3.00]
Filter alarms	Median [IQR]	0.15 [0.00, 0.50]	0.00 [0.00, 0.29]	0.18 [0.00, 0.50]	0.38 [0.00, 0.86]
TMP alarms	Median [IQR]	0.00 [0.00, 0.00]	0.00 [0.00, 0.00]	0.00 [0.00, 0.00]	0.00 [0.00, 0.00]
FP alarms	Median [IQR]	0.12 [0.00, 0.41]	0.00 [0.00, 0.20]	0.17 [0.00, 0.40]	0.29 [0.00, 0.67]
Clotting alarms	Median [IQR]	0.00 [0.00, 0.05]	0.00 [0.00, 0.00]	0.00 [0.00, 0.08]	0.00 [0.00, 0.12]
Fluid balance goal (mL/kg/day)	Median [IQR]	− 13.77 [− 20.04, − 8.25]	− 13.67 [− 21.72, − 5.22]	− 14.27 [− 18.44, − 9.78]	− 13.42 [− 19.82, − 8.20]
Net fluid balance (mL/kg/day)	Median [IQR]	3.01 [− 5.96, 17.11]	9.95 [− 3.29, 28.39]	0.71 [− 7.95, 11.90]	2.50 [− 5.18, 10.89]
FBgap (mL/kg/day)	Median [IQR]	17.37 [8.22, 30.39]	21.21 [10.23, 42.88]	14.49 [6.30, 25.49]	16.33 [9.30, 27.10]
Total TTL during entire CRRT (hours)	Median [IQR]	6.38 [1.65, 15.80]	0.94 [0.22, 3.00]	9.81 [4.45, 17.52]	14.12 [6.73, 28.61]
%TTL	Median [IQR]	8.14 [4.34, 12.45]	2.57 [1.20, 4.33]	8.14 [6.90, 9.48]	15.21 [12.48, 19.31]

%TTL, percent treatment time loss; ICU, intensive care unit; SOFA, sequential organ failure assessment; eGFR, estimated glomerular filtration rate; TMP, transmembrane pressure; FP, filter pressure; FBgap, fluid balance gap; IQR, interquartile range

*The tertile cutoffs for %TTL values are as follows: lowest: < 5.63%, middle: 5.63–10.76%, highest: ≥ 10.77%

†The number of each alarm is the average per day

**Table 2:** Clinical and process outcomes stratified by %TTL tertiles

		Overall	%TTL lowest tertile	%TTL middle tertile	%TTL highest tertile
Hospital mortality	*n* (%)	271 (54.2)	98 (58.7)	89 (53.6)	84 (50.3)
Length of hospital stay (days)	Median [IQR]	15 [7, 28]	12 [5, 24]	17 [9, 29]	16 [9, 30]
Length of ICU stay (days)	Median [IQR]	10 [5, 19]	6 [3, 13]	12 [7, 21]	12 [7, 21]
Total CRRT duration (days)	Median [IQR]	5 [3, 9]	4 [2, 6]	7 [4, 11]	5 [3, 11]
ICU-free days	Median [IQR]	0 [0, 4]	0 [0, 11]	0 [0, 0]	0 [0, 4]
CRRT-free days	Median [IQR]	0 [0, 14]	0 [0, 21]	0 [0, 12]	0 [0, 15]
MV-free days	Median [IQR]	0 [0, 14]	0 [0, 16]	0 [0, 6]	0 [0, 16]
Cardiac support device-free days	Median [IQR]	0 [0, 28]	0 [0, 28]	0 [0, 28]	0 [0, 28]

The outcome of free days indicates the number of days within the 28-day period after CRRT initiation during which there was no exposure to each respective factor. If the patient died within the 28 days, the number of free days is treated as 0

ICU, intensive care unit; CRRT, continuous renal replacement therapy; MV, mechanical ventilation; IQR, interquartile range

### Relationship between clinical and machine factors and %TTL

In adjusted models with %TTL as the dependent variable, the β coefficient was −0.05 (95% Confidence Interval [CI] −0.09 to −0.003) for age and −2.39 (95%CI −4.09 to −0.69) for MV use at CRRT initiation. The β coefficient for %FO was 0.18 (95%CI 0.10–0.25), for access alarms 1.61 (95%CI 1.25–1.98), for return alarms 0.51 (95%CI 0.14–0.88), and for filter pressure alarms 1.75 (95%CI 0.33–3.46) (Table 3). We found no statistical evidence for association of sex, race, CCI, SOFA score at CRRT initiation, use of cardiac support device at CRRT initiation, TMP alarms, and clotting alarms with %TTL.

**Table 3:** Association of patient and machine factors with %TTL

	β coefficient	95%CI
Age, per 1-year increase	− 0.05	− 0.09 to − 0.003
Male sex, vs. female	− 0.53	− 1.76 to 0.70
Black race, vs. other or unknown	1.46	− 3.09 to 6.00
White race, vs. other or unknown	− 0.03	− 4.05 to 3.99
CCI, per 1-point increase	0.14	− 0.08 to 0.36
SOFA score at CRRT initiation, per 1-point increase	0.18	− 0.02 to 0.37
MV at CRRT initiation	− 2.39	− 4.09 to − 0.69
Cardiac support device at CRRT initiation	− 0.88	− 2.94 to 1.18
%Fluid overload, per 1% increase	0.18	0.10 to 0.25
Access alarms, per 1-alarm increase	1.61	1.25 to 1.98
Return alarms, per 1-alarm increase	0.51	0.14 to 0.88
TMP alarms, per 1-alarm increase	− 1.22	− 4.34 to 1.89
FP alarms, per 1-alarm increase	1.75	0.03 to 3.46
Clotting alarms, per 1-alarm increase	3.46	− 2.11 to 9.03

Linear regression model with %TTL as the dependent variable as well as the factors shown in the table as independent variables was performed

%TTL, percent treatment time loss; CCI, Charlson comorbidity index; SOFA, sequential organ failure assessment; CRRT, continuous renal replacement therapy; MV, mechanical ventilation; TMP, transmembrane pressure; FP, filter pressure; FBgap, fluid balance gap; IQR, interquartile range

### Relationship between %TTL and FBgap

Based on FSA, a significant interaction between %TTL and the total number of alarms was observed, suggesting that the association of %TTL with FBgap is influenced by machine alarms. Therefore, considering the variation in the distribution of %TTL according to the number of alarms, we decided to categorize the total number of alarms into three groups (0–2, 3–5, and 6 + alarms per CRRT-day) for further analysis (Additional Fig. 3). The association of %TTL with FBgap varied across the total alarms subgroups (Fig. 2).

In adjusted models with the Bonferroni correction, there was a negative association between %TTL and FBgap without stratification by the total alarms subgroups (Table 4). However, a significant positive association was only found in the subgroup with a high number of alarms (≥ 6), with a β coefficient of 0.72 (95% CI 0.34 to 1.09) in the unadjusted model and a β coefficient of 0.87 (95% CI 0.48 to 1.26) in the adjusted model with the Bonferroni correction (Table 4).

**Table 4:** Association of %TTL (independent variable) with FBgap (dependent variable)

	β coefficient	95%CI	*p* value	Interaction between %TTL and total alarms
Model 1	− 0.49	− 0.80 to − 0.19	0.002^*^	Significant (*p* < 0.001)
Model 2	− 0.31	− 0.53 to − 0.09	0.006	–
Total alarms: 0–2	− 0.41	− 0.94 to 0.13	0.134	–
Total alarms: 3–5	− 0.06	− 0.35 to 0.22	0.665	–
Total alarms: 6 +	0.72	0.34 to 1.09	< 0.001^*^	–
Model 3	− 0.48	− 0.78 to − 0.18	0.002^*^	Significant (*p* < 0.001)
Model 4	− 0.26	− 0.49 to − 0.04	0.021	–
Total alarms: 0–2	− 0.37	− 0.88 to 0.14	0.157	–
Total alarms: 3–5	− 0.11	− 0.41 to 0.19	0.480	–
Total alarms: 6 +	0.87	0.48 to 1.26	< 0.001^*^	–

The β coefficient represents the change in FBgap for each 1% increase in %TTL

Model 1: A linear regression with FBgap as the dependent variable and %TTL as the independent variable, as well as the interaction term between %TTL and total alarms

Model 2: A linear regression with FBgap as the dependent variable and %TTL as the independent variable, without including the interaction term. In addition, a subgroup analysis was performed for each category of total alarms (0–2, 3–5, 6 +)

Model 3: A linear regression with FBgap as the dependent variable and %TTL as the main independent variable, including the interaction term between %TTL and total alarms. Other covariates included age, sex, race, CCI, SOFA score at CRRT initiation, use of MV at CRRT initiation, use of circulatory support devices (any of IABP, VAD, ECMO) at CRRT initiation, and %fluid overload as independent variables

Model 4: A linear regression with FBgap as the dependent variable and %TTL as the main independent variable, without including the interaction term. The covariates included the same as in Model 3. In addition, a subgroup analysis was performed for each category of total alarms (0–2, 3–5, 6 +)

CI, confidence interval; FBgap, fluid balance gap; %TTL, percent treatment time loss; CCI, Charlson comorbidity index; SOFA, sequential organ failure assessment; CRRT, continuous renal replacement therapy; MV, mechanical ventilation; IABP, intra-aortic balloon pump; VAD, ventricular assist device; ECMO, extracorporeal membrane oxygenation

^*^After applying the Bonferroni correction, the significance threshold was set to *p* < 0.005, as 10 models were used

### Relationship between %TTL and process outcomes

Adjusted models did not show statistical evidence for an association of %TTL with in-hospital mortality (odds ratio per 1% increase in %TTL: 0.99, 95% CI 0.96–1.03). Furthermore, we found no statistical evidence that %TTL was associated with ICU-free days (β coefficient: − 0.13, 95% CI − 0.27 to 0.003), CRRT-free days (β coefficient: − 0.11, 95% CI − 0.26 to 0.05), or MV-free days (β coefficient: − 0.10, 95% CI − 0.26 to 0.06) (Additional Table 5). In the survivor-only analysis, the estimates were as follows: ICU-free days (β coefficient: − 0.26, 95% CI − 0.49 to − 0.03), CRRT-free days (β coefficient: − 0.22, 95% CI − 0.47 to 0.04), and MV-free days (β coefficient: − 0.17, 95% CI − 0.44 to 0.09) (Additional Table 5).

## Discussion

We conducted a retrospective cohort study with the novel approach of combining EHR and CRRT machine data to investigate the relationship between %TTL (i.e. CRRT downtime) and FBgap (i.e. the gap between achieved and goal fluid balance) in critically ill adults. Against our hypothesis, %TTL was negatively associated with FBgap in the overall cohort (i.e. higher %TTL, lower FBgap). However, given the effect modification of total number of alarms on the relationship of %TTL with FBgap, the only statistically significant relationship that prevailed in stratified analyses was that higher %TTL was positively associated with FBgap in patients with ≥ 6 alarms per CRRT-day. This suggests that downtime has a stronger impact on fluid management in patients with frequent alarms. One possible explanation for this is that downtime unrelated to alarms are more likely to be anticipated (ex. planned filter change for off unit procedure) and later compensated for clinically. Conversely, when the number of alarms is high, significant treatment time loss suggests that downtime is unexpected, leading to greater deviations in fluid management.

Prior literature has described some contributing factors to CRRT downtime, such as filter clotting, catheter malfunction, procedures, transfers, and patient mobilization [11, 14, 19]. Predictable downtimes may allow for adjustments in CRRT dose and goal setting, while unexpected issues like clotting or access failure may significantly impact CRRT deliverables, including fluid management. Future investigations should describe factors contributing to downtime, distinguishing between predictable and unpredictable factors. By doing so, we can aim to minimize unexpected factors and the downtime they cause, potentially leading to more optimal CRRT delivery.

The total downtime in this study was about 6.4 h over the entire CRRT course. Prior literature reports downtime in hours per day anywhere from 3.0 to 5.3 h. Compared to previous studies our %TTL was lower (8.14% vs. 22%) [11–14]. Downtime may vary significantly between study sites as different institutions may have different CRRT protocols, policies, and staffing. Furthermore, this study used data directly extracted from the CRRT machine to quantify downtime. This may have made this study more accurate in determining downtime and running time of CRRT, possibly resulting in the identified differences, and thus improving external validity of this study.

This study also noted %TTL was not associated with hospital mortality or process outcomes. Similar to our results, a prior retrospective observational study compared clinical outcomes between patients with < 20% downtime (*n* = 88) to those with > 20% downtime (*n* = 44). Despite some differences in clinical indicators, such as blood urea nitrogen and serum creatinine, they found no significant difference in 28-day mortality [14]. This study also did not find any relationship between %TTL and process outcomes (ex. MV or ICU free days). Although the 2019 ADQI consensus recommended a goal of < 10% downtime as a reasonable quality improvement measure, validation of proposed cutoffs is needed [10].

This study has many strengths including a large sample size with data from 500 patients with over 3,000 CRRT patient-days. In addition, this study introduces novelty in utilizing multimodal data—both EHR and CRRT machine data. Because we used data directly extracted from the CRRT machine our results may reflect higher accuracy when determining CRRT downtime. Nonetheless, this study also has limitations. First, it is a single center observational study. This study involved a population of about 90% white patients limiting extrapolation to other populations. We only accounted for select high yield clinical and machine variables, and therefore residual confounding by unmeasured variables is possible. It relied heavily on matching algorithms between EHR and CRRT machine data, which poses potential systematic errors in data mining. We attempted to overcome this by including only those patients with > 90% multimodal data matches. We also performed a 10% manual validation with the goal of identifying a systematic error if one existed. Also innate to our methodology of utilizing EHR data, some important data were unable to be systematically incorporated. For example, pre-ICU fluid resuscitation data were not available. For this reason, our %FO at CRRT initiation may not accurately reflect the fluid status of every patient. We also do not have data on etiology of CRRT downtime beyond alarm information. Since we were unable to obtain data on the specific indications for CRRT, the study population includes patients that received CRRT for purposes other than fluid management. Finally, while we followed previous research by focusing on the overall FB gap during the entire CRRT period [9], this approach may overlook the fluctuations in daily gaps. We are considering evaluating the trajectory of FB gap in future research. Relatedly, determining fluid needs, especially in a critically ill population, are often imprecise and frequently changing. Fluid balance goals may be prone to variability and subjectivity and are typically not guided by evidence or consensus which limits the reliability of FB gap as an established CRRT metric.

Despite these limitations, this study lays the groundwork for future work which should look at better assessment of patient fluid balance and more accurate fluid balance prescriptions. Additional research should be dedicated to elucidating specific causes of downtime and the clinical impact of each type as well as precision medicine-based approaches that decrease unnecessary alarms and downtime during CRRT. This study should also be replicated across other centers and other populations.

## Conclusion

This study found the association between %TTL and FBgap was significant only in patients with a high number of CRRT alarms (≥ 6 alarms per CRRT-day). Although this finding suggests that downtime due to unexpected factors (i.e. treatment interruptions due to recurrent alarms) may have a stronger impact on fluid management, the mechanisms behind this effect modification are not fully explored, and further studies are needed to understand the clinical implications. This study did not find an association between %TTL and hospital mortality or other process outcomes, such as ICU-free days, CRRT-free days, or MV-free days. This lack of association raises questions about the significance of %TTL as a clinical rather than a quality indicator of CRRT.

## Supplementary Information

Additional file 1.

## Data Availability

The data sets used and/or analyzed during the current study are available from the corresponding author on reasonable request.

## Abbreviations

ADQI: Acute Disease Quality Initiative
AKI: Acute kidney injury
CCI: Charlson comorbidity index
CI: Confidence interval
CRRT: Continuous renal replacement therapy
ECMO: Extracorporeal membrane oxygenation
eGFR: Estimated glomerular filtration rate
EHR: Electronic health record
FBgap: Fluid balance gap
FP: Filter pressure
%FO: Fluid overload
FSA: Feasible Solution Algorithm
IABP: Intra-aortic balloon pump
ICU: Intensive care unit
IQR: Interquartile range
IRB: Institutional review board
MV: Mechanical ventilation
SOFA: Sequential Organ Failure Assessment
TMP: Transmembrane pressure
%TTL: Percent treatment time loss
VAD: Ventricular assist device

## References

[1] Hoste EA, Bagshaw SM, Bellomo R, Cely CM, Colman R, Cruz DN, et al. Epidemiology of acute kidney injury in critically ill patients: the multinational AKI-EPI study. Intensive Care Med. 2015;41(8):1411–23. 26162677 10.1007/s00134-015-3934-7

[2] Neyra JA, Goldstein SL. Optimizing renal replacement therapy deliverables through multidisciplinary work in the intensive care unit. Clin Nephrol. 2018;90(1):1–5. 29633700 10.5414/CN109447

[3] Malbrain ML, Marik PE, Witters I, Cordemans C, Kirkpatrick AW, Roberts DJ, Van Regenmortel N. Fluid overload, de-resuscitation, and outcomes in critically ill or injured patients: a systematic review with suggestions for clinical practice. Anaesthesiol Intensive Ther. 2014;46(5):361–80. 25432556 10.5603/AIT.2014.0060

[4] Messmer AS, Zingg C, Müller M, Gerber JL, Schefold JC, Pfortmueller CA. Fluid overload and mortality in adult critical care patients-a systematic review and meta-analysis of observational studies. Crit Care Med. 2020;48(12):1862–70. 33009098 10.1097/CCM.0000000000004617

[5] Salahuddin N, Sammani M, Hamdan A, Joseph M, Al-Nemary Y, Alquaiz R, et al. Fluid overload is an independent risk factor for acute kidney injury in critically Ill patients: results of a cohort study. BMC Nephrol. 2017;18(1):45. 28143505 10.1186/s12882-017-0460-6 PMC5286805

[6] Vincent JL, Sakr Y, Sprung CL, Ranieri VM, Reinhart K, Gerlach H, et al. Sepsis in European intensive care units: results of the SOAP study. Crit Care Med. 2006;34(2):344–53. 16424713 10.1097/01.ccm.0000194725.48928.3a

[7] Woodward CW, Lambert J, Ortiz-Soriano V, Li Y, Ruiz-Conejo M, Bissell BD, et al. Fluid overload associates with major adverse kidney events in critically ill patients with acute kidney injury requiring continuous renal replacement therapy. Crit Care Med. 2019;47(9):e753–60. 31162196 10.1097/CCM.0000000000003862

[8] Neyra JA, Yessayan L, Thompson Bastin ML, Wille KM, Tolwani AJ. How to prescribe and troubleshoot continuous renal replacement therapy: a case-based review. Kidney. 2021;2(2):371–84. 10.34067/KID.0004912020 PMC8741005 35373031

[9] Neyra JA, Lambert J, Ortiz-Soriano V, Cleland D, Colquitt J, Adams P, et al. Assessment of prescribed vs. achieved fluid balance during continuous renal replacement therapy and mortality outcome. PLoS One. 2022;17(8):e0272913. 36006963 10.1371/journal.pone.0272913 PMC9409548

[10] Rewa OG, Tolwani A, Mottes T, Juncos LA, Ronco C, Kashani K, et al. Quality of care and safety measures of acute renal replacement therapy: workgroup statements from the 22nd acute disease quality initiative (ADQI) consensus conference. J Crit Care. 2019;54:52–7. 31349160 10.1016/j.jcrc.2019.07.003

[11] Fealy N, Baldwin I, Bellomo R. The effect of circuit “down-time” on uraemic control during continuous veno-venous haemofiltration. Crit Care Resusc. 2002;4(4):266–70. 16573439

[12] Uchino S, Fealy N, Baldwin I, Morimatsu H, Bellomo R. Continuous is not continuous: the incidence and impact of circuit “down-time” on uraemic control during continuous veno-venous haemofiltration. Intensive Care Med. 2003;29(4):575–8. 12577144 10.1007/s00134-003-1672-8

[13] Davies H, Leslie GD, Morgan D. A retrospective review of fluid balance control in CRRT. Aust Crit Care. 2017;30(6):314–9. 27338750 10.1016/j.aucc.2016.05.004

[14] Shin J, Song HC, Hwang JH, Kim SH. Impact of downtime on clinical outcomes in critically Ill patients with acute kidney injury receiving continuous renal replacement therapy. ASAIO J. 2022;68(5):744–52. 34506331 10.1097/MAT.0000000000001549

[15] Cook RD. Detection of influential observation in linear regression. Technometrics. 1977;19(1):15.

[16] Vincent JL, Moreno R, Takala J, Willatts S, De Mendonça A, Bruining H, et al. The SOFA (Sepsis-related Organ Failure Assessment) score to describe organ dysfunction/failure. On behalf of the working group on sepsis-related problems of the european society of intensive care medicine. Intensive Care Med. 1996;22(7):707–10. 8844239 10.1007/BF01709751

[17] Quan H, Sundararajan V, Halfon P, Fong A, Burnand B, Luthi JC, et al. Coding algorithms for defining comorbidities in ICD-9-CM and ICD-10 administrative data. Med Care. 2005;43(11):1130–9. 16224307 10.1097/01.mlr.0000182534.19832.83

[18] Lambert J, Gong L, Elliott CF, Thompson K, Stromberg A. rFSA: an R package for finding best subsets and interactions. R J. 2018;10(2):295–308. 35719742 10.32614/rj-2018-059 PMC9205535

[19] Baldwin I. Factors affecting circuit patency and filter “life.” Contrib Nephrol. 2007;156:178–84. 17464125 10.1159/000102081

