# Supplementary material for: Association of continuous renal replacement therapy downtime with fluid balance gap and clinical outcomes: a retrospective cohort analysis utilizing EHR and machine data
Source: J Intensive Care. 2024 Dec 31;12:55. doi: 10.1186/s40560-024-00772-w (PMC11686856; doi:10.1186/s40560-024-00772-w)
Supplement: Supplementary file 1 — Additional file 1. [file 40560_2024_772_MOESM1_ESM.docx]

**Additional Materials**

**Additional Table 1: STROBE Checklist**

**Additional Figure 1: Distribution of FBgap**

**Additional Table 2: Details in Machine Data Extraction and Process**

**Additional Figure 2: Density plot for distribution of fluid balance goal and net fluid balance**

**Additional Table 3: Cohort characteristics stratified by hospital death**

**Additional Table 4: Association of patient and machine factors with FBgap**

**Additional Figure 3: Box plot for distribution of %TTL stratified by number of total alarms**

**Additional Table 5: Association of %TTL with hospital mortality and process outcomes**

**in the entire cohort and survivors only**

**Additional Table 1: STROBE Checklist**

|  | | Item No | Recommendation | Page No |
| --- | --- | --- | --- | --- |
| **Title and abstract** | | 1 | (*a*) Indicate the study’s design with a commonly used term in the title or the abstract | 1 |
|  |  |  | (*b*) Provide in the abstract an informative and balanced summary of what was done and what was found | 2 |
| Introduction | | | | |
| Background/rationale | | 2 | Explain the scientific background and rationale for the investigation being reported | 3 |
| Objectives | | 3 | State specific objectives, including any prespecified hypotheses | 3 |
| Methods | | | | |
| Study design | | 4 | Present key elements of study design early in the paper | 4 |
| Setting | | 5 | Describe the setting, locations, and relevant dates, including periods of recruitment, exposure, follow-up, and data collection | 4 |
| Participants | | 6 | (*a*) Give the eligibility criteria, and the sources and methods of selection of participants. Describe methods of follow-up | 4 |
|  |  |  | (*b*) For matched studies, give matching criteria and number of exposed and unexposed | N/A |
| Variables | | 7 | Clearly define all outcomes, exposures, predictors, potential confounders, and effect modifiers. Give diagnostic criteria, if applicable | 5, 6 and Sup. Table 2 |
| Data sources/ measurement | | 8* | For each variable of interest, give sources of data and details of methods of assessment (measurement). Describe comparability of assessment methods if there is more than one group | 4 and 5 |
| Bias | | 9 | Describe any efforts to address potential sources of bias | 4, 5, 6, 7 |
| Study size | | 10 | Explain how the study size was arrived at | Fig 1 |
| Quantitative variables | | 11 | Explain how quantitative variables were handled in the analyses. If applicable, describe which groupings were chosen and why | 5 and 6 |
| Statistical methods | | 12 | (*a*) Describe all statistical methods, including those used to control for confounding | 6 and 7 |
|  |  |  | (*b*) Describe any methods used to examine subgroups and interactions | 6 |
|  |  |  | (*c*) Explain how missing data were addressed | 4 |
|  |  |  | (*d*) If applicable, explain how loss to follow-up was addressed | N/A |
|  |  |  | (*e*) Describe any sensitivity analyses | N/A |
| Results | | | |  |
| Participants | | 13* | (a) Report numbers of individuals at each stage of study—eg numbers potentially eligible, examined for eligibility, confirmed eligible, included in the study, completing follow-up, and analysed | Fig 1 |
|  |  |  | (b) Give reasons for non-participation at each stage | Fig 1 |
|  |  |  | (c) Consider use of a flow diagram | Fig 1 |
| Descriptive data | | 14* | (a) Give characteristics of study participants (eg demographic, clinical, social) and information on exposures and potential confounders | 7, 8 and Table 1 |
|  |  |  | (b) Indicate number of participants with missing data for each variable of interest | Fig 1 |
|  |  |  | (c) Summarise follow-up time (eg, average and total amount) | Table 1 and 2 |
| Outcome data | | 15* | Report numbers of outcome events or summary measures over time | 7, 8 and Table 2 |
| Main results | 16 | (*a*) Give unadjusted estimates and, if applicable, confounder-adjusted estimates and their precision (eg, 95% confidence interval). Make clear which confounders were adjusted for and why they were included | | 7,8, 9 |
|  |  | (*b*) Report category boundaries when continuous variables were categorized | | 7,8, 9 |
|  |  | (*c*) If relevant, consider translating estimates of relative risk into absolute risk for a meaningful time period | | N/A |
| Other analyses | 17 | Report other analyses done—eg analyses of subgroups and interactions, and sensitivity analyses | | 8, 9 |
| Discussion | | | | |
| Key results | 18 | Summarise key results with reference to study objectives | | 9, 10 |
| Limitations | 19 | Discuss limitations of the study, taking into account sources of potential bias or imprecision. Discuss both direction and magnitude of any potential bias | | 11 |
| Interpretation | 20 | Give a cautious overall interpretation of results considering objectives, limitations, multiplicity of analyses, results from similar studies, and other relevant evidence | | 8-11 |
| Generalisability | 21 | Discuss the generalisability (external validity) of the study results | | 11 |
| Other information | | | | |
| Funding | 22 | Give the source of funding and the role of the funders for the present study and, if applicable, for the original study on which the present article is based | | 12 |

**
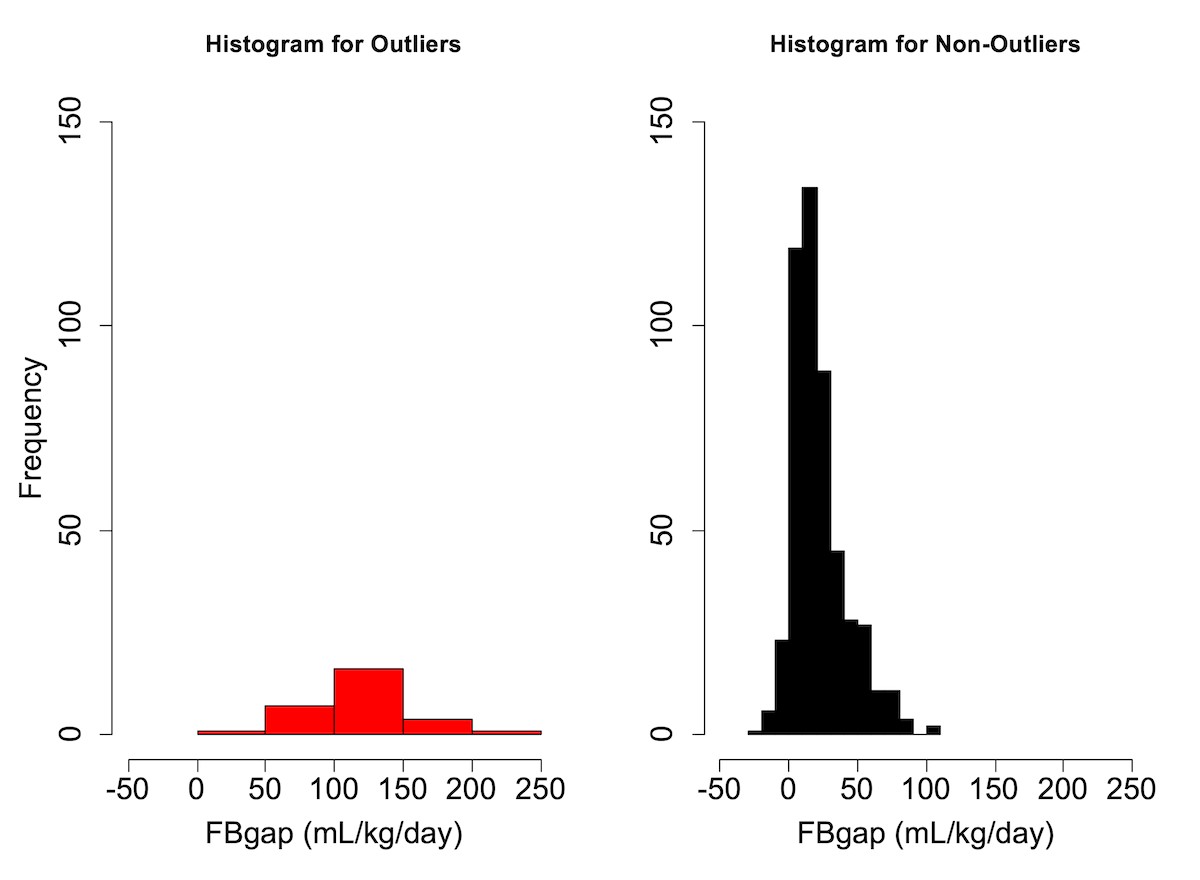
Additional Figure 1: Distribution of FBgap**

FBgap, fluid balance gap

**Additional Table 2: Details of Data Extraction, Matching, and Alarm Processing Procedures**

| **Automated Sweep** | Export Prismaflex data including 'Patient ID' (MD5 hash). |
| --- | --- |
|  | Truncate MD5 hash to 20 characters for matching, as Prismaflex outputs truncated hashes. |
|  | Use a list of patient identifiers (MRN, Encounter IDs, Names) to match data. |
|  | Try multiple formats of patient identifiers against the truncated MD5 hashes to find matches. |
| **Special Cases** | Manually match files with no patient identifier. |
|  | Apply brute force approach to test all 9-digit MRNs, which helped to match additional records not found in the initial EHR data |
| **Manual Sweep** | Organize matched patient files into specific folders. |
|  | Keep unmatched data in a separate folder for further analysis. |
| **Fluid Removal Data** | Match patient files with daily fluid removal data computed from EHR data. Assume correctness of data within ICU date range unless there are time conflicts. |
| **Validation** | Include overlapping files from the same treatment to ensure completeness. |
|  | Cross-check fluid balance trajectories and other data points (e.g., weight) between machine data and EHR data for additional validation. |
| **Duplicate Removal** | Check patient folders for duplicate CRRT files. Retain files matching within +/- 1 day of CRRT start and stop dates; delete non-matching data. |
| **Alarm Identification** | Identify alarms based on the machine's threshold settings for measured pressures. Whenever these pressures exceeded a specified numeric threshold, an alarm was triggered.  To avoid counting repeated alarms that do not represent unique issues, any alarms of the same category occurring within 5 minutes of a preceding alarm were not counted as new alarms. Pressure oscillations can cause the machine to alarm repeatedly within a short period, which does not necessarily indicate a unique issue. |

MRN- medical record number, EHR- electronic health record, ICU- intensive care unit, CRRT- continuous renal replacement therapy

**Additional Figure 2: Density plot for distribution of fluid balance goal and net fluid balance**


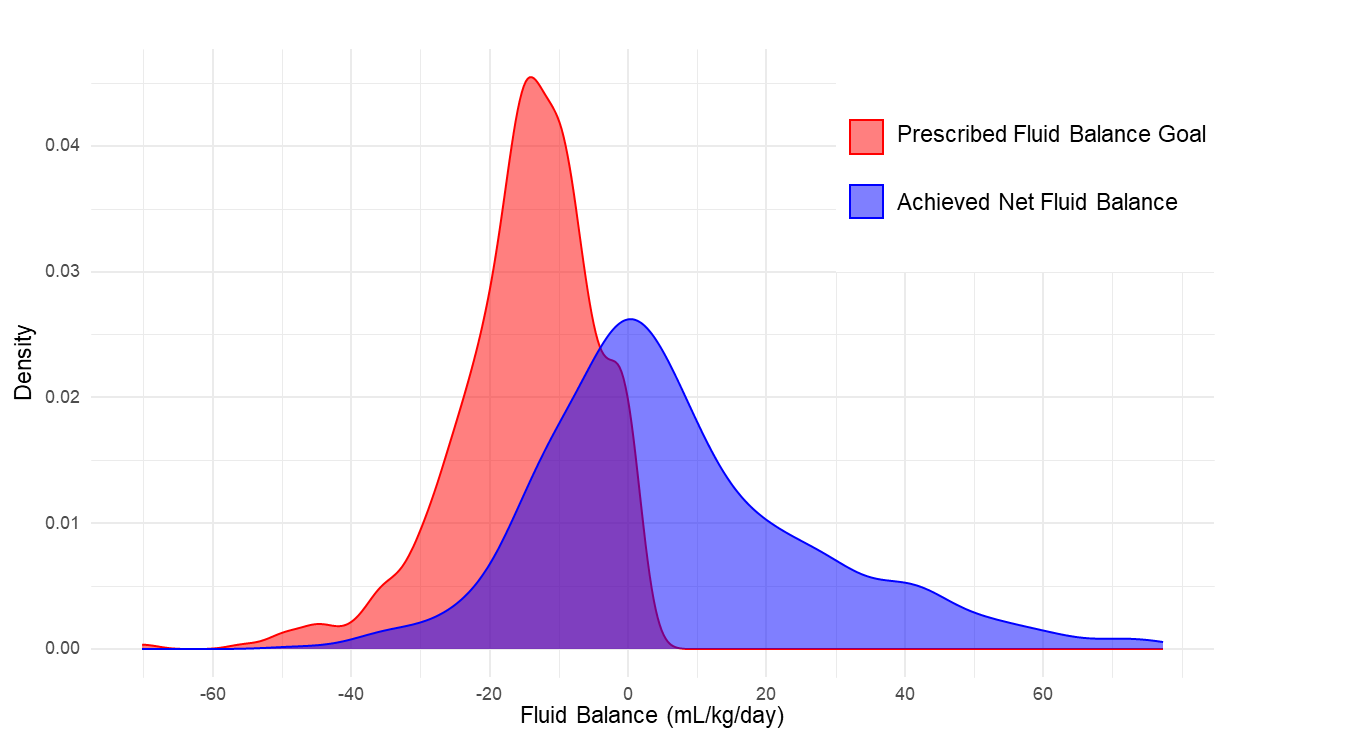


**Additional Table 3: Cohort characteristics stratified by hospital mortality**

| **Patient factors** | | |  | **Survived** | **Expired** |
| --- | --- | --- | --- | --- | --- |
|  | Age (years) | | median [IQR] | 58.0 [47.0, 66.0] | 62.0 [52.5, 69.0] |
|  | Sex, male | | n (%) | 135 (59.0) | 170 (62.7) |
|  | Race | | n (%) |  |  |
|  |  | Black |  | 0 (0.0) | 12 (4.4) |
|  |  | White |  | 24 (10.5) | 15 (5.5) |
|  |  | Other or unknown |  | 205 (89.5) | 244 (90.0) |
|  | Body weight at ICU admission (kg) | | median [IQR] | 93.7 [76.0, 110.2] | 93.6 [80.9, 114.4] |
|  | Charlson comorbidity index | | median [IQR] | 5 [3, 6] | 5 [2, 7] |
|  | SOFA score at CRRT initiation | | median [IQR] | 12 [10, 15] | 14 [12, 16] |
|  | SOFA score at ICU admission | | median [IQR] | 11 [8, 14] | 12 [9, 15] |
|  | Baseline eGFR (mL/min/1.73m2) | | median [IQR] | 30.9 [14.2, 65.7] | 50.7 [22.4, 76.8] |
|  | Mechanical ventilation at CRRT initiation | | n (%) | 170 (74.2) | 234 (86.3) |
|  | Cardiac support device at CRRT initiation | | n (%) | 15 (6.6) | 37 (13.7) |
|  | Time from ICU admission to CRRT initiation | | median [IQR] | 1 [0, 4] | 1 [1, 4] |
|  | %Fluid overload from ICU admission to CRRT initiation | | median [IQR] | 0.92 [0, 4.82] | 0.92 [0, 4.55] |
| **Machine factors*** | | |  |  |  |
|  | Total alarms | | median [IQR] | 3.30 [1.67, 5.55] | 2.40 [1.33, 4.00] |
|  | Catheter alarms | | median [IQR] | 3.00 [1.50, 5.10] | 2.00 [1.00, 3.50] |
|  |  | Access alarms | median [IQR] | 1.53 [0.80, 2.60] | 1.00 [0.39, 2.00] |
|  |  | Return alarms | median [IQR] | 1.00 [0.44, 2.11] | 1.00 [0.43, 1.52] |
|  | Filter alarms | | median [IQR] | 0.20 [0.00, 0.50] | 0.11 [0.00, 0.50] |
|  |  | TMP alarms | median [IQR] | 0.00 [0.00, 0.00] | 0.00 [0.00, 0.00] |
|  |  | FP alarms | median [IQR] | 0.15 [0.00, 0.40] | 0.08 [0.00, 0.42] |
|  |  | Clotting alarms | median [IQR] | 0.00 [0.00, 0.08] | 0.00 [0.00, 0.00] |
|  | Fluid balance goal (mL/kg/day) | | median [IQR] | -15.02 [-20.62, -10.10] | -12.32 [-19.02, -5.83] |
|  | Net fluid balance (mL/kg/day) | | median [IQR] | -2.92 [-11.49, 4.26] | 11.52 [1.08, 28.05] |
|  | FBgap (mL/kg/day) | | median [IQR] | 11.52 [5.22, 20.26] | 23.67 [13.07, 41.88] |
|  | Total TTL during entire CRRT (hours) | | median [IQR] | 8.51 [3.11, 21.01] | 4.91 [1.07, 12.21] |
|  | %TTL | | median [IQR] | 8.81 [4.87, 12.91] | 7.74 [3.90, 11.73] |

ICU, intensive care unit; SOFA, sequential organ failure assessment; eGFR, estimated glomerular filtration rate; TMP, transmembrane pressure; FP, filter pressure; FBgap, fluid balance gap; IQR, interquartile range

***** The number of each alarm is the average per day.

**Additional Table 4: Association of patient and machine factors with FBgap**

|  | **Overall** | | **0 - 2 alarms/day** | | **3 -5 alarms/day** | | **6+ alarms/day** | |
| --- | --- | --- | --- | --- | --- | --- | --- | --- |
|  | **β coefficient** | **95%CI** | **β coefficient** | **95%CI** | **β coefficient** | **95%CI** | **β coefficient** | **95%CI** |
| %TTL, per 1% increase | -0.27 | -0.50 to -0.04 | -0.35 | -0.86 to 0.16 | -0.13 | -0.44 to 0.18 | 0.89 | 0.49 to 1.29 |
| Age, per 1-year increase | 0.07 | -0.06 to 0.19 | 0.00 | -0.23 to 0.24 | 0.05 | -0.11 to 0.22 | 0.04 | -0.15 to 0.24 |
| Male sex, vs.female | -1.05 | -4.55 to 2.44 | 1.96 | -4.47 to 8.38 | -0.54 | -5.36 to 4.29 | -2.81 | -8.23 to 2.62 |
| Black race,  vs. other or unknown | 5.19 | -7.65 to 18.04 | 12.24 | -9.22 to 33.71 | 10.96 | -9.73 to 31.65 | -6.03 | -26.47 to 14.42 |
| White race,  vs. other or unknown | 1.86 | -9.54 to 13.25 | 7.16 | -11.28 to 25.60 | 5.35 | -13.43 to 24.13 | 0.18 | -18.77 to 19.13 |
| CCI,  per 1-point increase | -0.20 | -0.82 to 0.43 | -0.72 | -1.95 to 0.51 | -0.23 | -1.07 to 0.62 | 0.61 | -0.30 to 1.52 |
| SOFA score at CRRT initiation,  per 1-point increase | 1.49 | 0.93 to 2.04 | 2.26 | 1.17 to 3.36 | 1.37 | 0.63 to 2.11 | 0.01 | -0.79 to 0.81 |
| MV at CRRT initiation | 2.27 | -2.52 to 7.07 | 5.22 | -4.00 to 14.44 | -3.36 | -9.95 to 3.22 | 8.08 | 1.19 to 14.97 |
| Cardiac support device  at CRRT initiation | -3.85 | -9.63 to 1.94 | -3.18 | -13.92 to 7.55 | -4.41 | -11.66 to 2.83 | -0.93 | -12.54 to 10.69 |
| %Fluid overload, per 1% increase | -0.07 | -0.27 to 0.13 | -0.29 | -0.71 to 0.14 | -0.01 | -0.26 to 0.23 | -0.15 | -0.49 to 0.18 |

FBgap, fluid balance gap; %TTL, percent treatment time loss; CCI, Charlson comorbidity index; SOFA, sequential organ failure assessment; CRRT, continuous renal replacement therapy; MV, mechanical ventilation; CI, confidence interval

Linear regression models with FBgap as the dependent variable as well as the factors shown in the table as the independent variables were performed.

**Additional Figure 3: Distribution of %TTL stratified by number of total alarms**


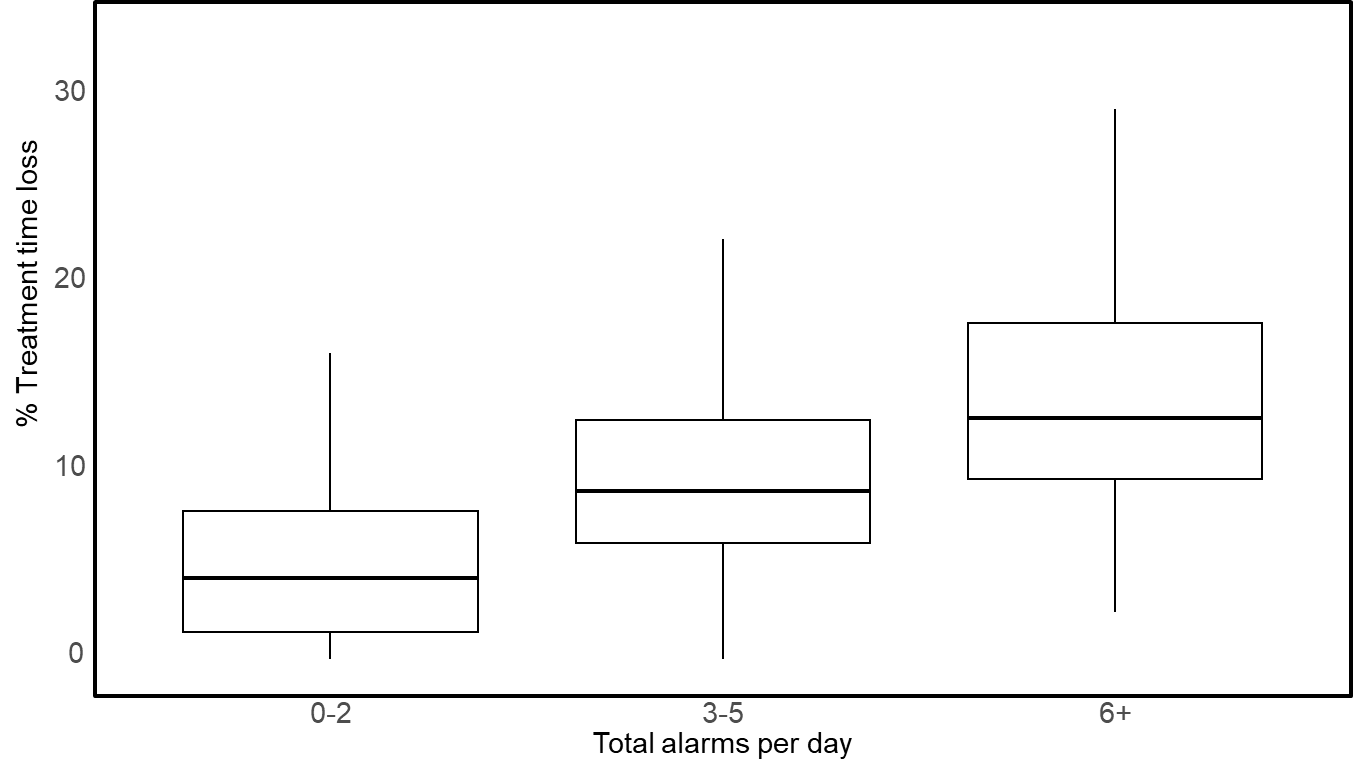


%TTL, percent treatment time loss

**Additional Table 5: Association of %TTL with hospital mortality and process outcomes in the entire cohort and survivors only**

|  |  | **Hospital mortality** | | **ICU-free days** | | **CRRT-free days** | | **MV-free days** | |
| --- | --- | --- | --- | --- | --- | --- | --- | --- | --- |
| **Entire cohort** | | **OR** | **95%CI** | **β coefficient** | **95%CI** | **β coefficient** | **95%CI** | **β coefficient** | **95%CI** |
| Overall | | 0.99 | 0.96 to 1.03 | -0.13 | -0.27 to 0.003 | -0.11 | -0.26 to 0.05 | -0.10 | -0.26 to 0.06 |
|  | 0 - 2 alarms/day | 0.99 | 0.94 to 1.04 | -0.11 | -0.31 to 0.08 | -0.11 | -0.33 to 0.10 | -0.11 | -0.33 to 0.11 |
|  | 3 -5 alarms/day | 1.00 | 0.96 to 1.05 | -0.12 | -0.26 to 0.03 | -0.1 | -0.27 to 0.06 | -0.07 | -0.25 to 0.11 |
|  | 6+ alarms/day | 1.05 | 0.98 to 1.12 | -0.03 | -0.30 to 0.25 | -0.12 | -0.44 to 0.20 | -0.12 | -0.45 to 0.21 |
| **Survivor only** | |  |  |  |  |  |  |  |  |
| Overall | | - | - | -0.26 | -0.49 to -0.03 | -0.22 | -0.47 to 0.04 | -0.17 | -0.44 to 0.09 |
|  | 0 - 2 alarms/day | - | - | -0.15 | -0.50 to 0.19 | -0.14 | -0.52 to 0.24 | -0.16 | -0.54 to 0.23 |
|  | 3 -5 alarms/day | - | - | -0.33 | -0.61 to -0.05 | -0.3 | -0.60 to 0.01 | -0.23 | -0.56 to 0.11 |
|  | 6+ alarms/day | - | - | 0.21 | -0.23 to 0.64 | 0.17 | -0.27 to 0.61 | 0.16 | -0.32 to 0.64 |

FBgap, fluid balance gap; %TTL, percent treatment time loss; CCI, Charlson comorbidity index; SOFA, sequential organ failure assessment; CRRT, continuous renal replacement therapy; MV, mechanical ventilation; ICU, intensive care unit; OR, odds ratio; CI, confidence interval

Logistic and linear regression models with each clinical outcome as the dependent variable and %TTL as the main independent variable. Other covariates included age, sex, race, CCI, SOFA score at CRRT initiation, use of MV at CRRT initiation, use of circulatory support devices (any of IABP, VAD, ECMO) at CRRT initiation, and %fluid overload as independent variables. The models for the overall cohort and overall survivors also included the interaction term between %TTL and total alarms, but these were all insignificant (*p* > 0.1).

The outcome of free days indicates the number of days within the 28-day period after CRRT initiation during which there was no exposure to each respective factor. If the patient died within the 28 days, the number of free days is treated as 0.
